# No Effect of Transcranial Direct Current Stimulation of the Auditory Cortex on Auditory-Evoked Potentials

**DOI:** 10.3389/fnins.2018.00880

**Published:** 2018-11-27

**Authors:** Katharina Kunzelmann, Lea Meier, Matthias Grieder, Yosuke Morishima, Thomas Dierks

**Affiliations:** Division of Systems Neuroscience of Psychopathology, Translational Research Center, University Hospital of Psychiatry Bern, Bern, Switzerland

**Keywords:** auditory-evoked potential, event-related potential, electroencephalography, transcranial direct current stimulation, non-invasive brain stimulation, P50, N100, P200

## Abstract

Transcranial direct current stimulation (tDCS) is a non-invasive brain stimulation technique to change cortical excitability. Its effects are shown for cognitive processing, and behavior in the motor and perceptual domains. However, evidence of tDCS effects in the perceptual domain particularly for auditory processing is rare. Therefore, and in the context of disturbances in auditory processing in psychiatric populations, e.g., in patients with auditory verbal hallucinations, we aimed to investigate the potential modulatory effect of tDCS on the excitability of left posterior temporal cortex in detail. We included 24 healthy participants in a crossover design, applying sham and anodal stimulation in two measurement sessions 1 week apart. Electroencephalography (EEG) was recorded while participants listened to tones before, during, and after stimulation. Amplitudes and latencies of P50, N100, and P200 auditory-evoked potentials (AEP) were compared between anodal and sham stimulation, and between time points before, during, and after tDCS. In contrast to previous studies, results demonstrate no significant differences between stimulation types or time points for any of the investigated AEP amplitudes or latencies. Furthermore, a topographical analysis did not show any topographical differences during peak time periods of the investigated AEP for stimulation types and time points besides a habituation effect. Thus, our results suggest that tDCS modulation of excitability of the left posterior temporal cortex, targeting the auditory cortex, does not have any effect on AEP. This is particularly interesting in the context of tDCS as a potential treatment for changed electrophysiological parameters and symptoms of psychiatric diseases, e.g., lower N100 or auditory verbal hallucinations in schizophrenia.

## Introduction

Transcranial direct current stimulation (tDCS) is a non-invasive brain stimulation technique to change cortical excitability (Nitsche et al., 2008). For tDCS, a low electrical current is applied through two or more electrodes placed on the scalp. Anodal stimulation with the anode considered as “active” electrode is supposed to increase excitability of stimulated regions by depolarization of neurons. In contrast, cathodal stimulation with the cathode considered as “active” electrode is assumed to decrease excitability by hyperpolarization of stimulated neurons (Nitsche et al., 2008). However, a recent meta-analytical review revealed that effects of stimulation depend on the domain of investigation, i.e., motor domain or “cognition” (category of all non-motor domain studies compiled by the authors), and the polarity of the active electrode (Jacobson et al., 2012). The authors conclude that increases in excitability after anodal stimulation and especially decreases in excitability after cathodal stimulation are more reliably shown in the motor domain. Furthermore, research on effects of tDCS in the “cognitive” domain demonstrates reliable increase of excitability after anodal stimulation but no reliable decrease of excitability by cathodal stimulation (Jacobson et al., 2012).

Besides effects on cognition (Antal et al., 2014) and behavior in the motor domain (Jacobson et al., 2012), tDCS was also shown to modulate behavior in the perceptual domain, i.e., modulation of visual (Antal et al., 2011; Costa et al., 2015), somato-sensory (Costa et al., 2015), and auditory processing (Costa et al., 2015; Heimrath et al., 2016a). Regarding auditory processing, application of tDCS affected performance in a task assessing temporal resolution of auditory processing (Ladeira et al., 2011; Heimrath et al., 2014). Further, tDCS also changed performance in pitch memory (Vines et al., 2006; Schaal et al., 2013), pitch matching (Loui et al., 2010), and pitch discrimination (Mathys et al., 2010; Matsushita et al., 2015). In addition to behavioral performance, tDCS effects on electrophysiological changes for auditory discrimination were investigated using Mismatch Negativity (MMN). MMN is an increased negative event-related potential of a “deviant” stimulus, which deviates from standard stimuli in frequency, duration, or pitch, in an oddball paradigm (Naatanen et al., 1978, 2007; Naatanen and Michie, 1979). MMN amplitudes for frequency deviants were reduced after anodal compared to sham and cathodal stimulation of the right inferior frontal cortex (Chen et al., 2014), and the bilateral auditory cortex (AC) (Heimrath et al., 2015). In contrast, two pilot studies which included 12 subjects each report opposite effects of increased MMN amplitudes after anodal stimulation of the left AC compared to baseline (Impey and Knott, 2015; Impey et al., 2016), and decreased amplitudes after cathodal stimulation of the left AC compared to baseline (Impey et al., 2016). However, it is difficult to disentangle tDCS effects on MMN, because tDCS may modulate responses to both “deviant” and “standard” stimuli. Furthermore, the small sample size in both studies, and the subdivision into two groups based on performance in one of them, call for caution in the interpretation of these results. Zaehle et al. (2011) aimed to investigate the impact of tDCS on excitability of the AC by evaluating auditory-evoked potentials (AEP) after tone presentation. They applied 11 min of either anodal, cathodal, or sham tDCS at 1.25 mA of current intensity with the active electrode either located temporally (over T7 of the 10–20 EEG system) or temporo-parietally (over CP5). Their results demonstrate changes in some of the typical components occurring after tone presentation. In particular, after anodal compared to sham and cathodal stimulation at the temporal location, Zaehle et al. (2011) found an increase in the amplitude of P50, a positive potential occurring about 50 ms after stimulus onset. Further, P50 amplitude was higher for anodal stimulation over the temporal compared to the temporo-parietal location and lower for cathodal stimulation over the temporal compared to the temporo-parietal location. Amplitudes for the N100, a negative potential about 100 ms after stimulus onset (Picton et al., 1974), were lower for cathodal compared to sham and anodal stimulation over the temporo-parietal location. Placing the cathode temporally resulted in lower N100 amplitudes compared to placing it temporo-parietally. No effects of tDCS were observed for P50 latencies but N100 latencies were shorter after anodal compared to sham stimulation temporo-parietally. Zaehle et al. (2011) did not report changes in any other of the auditory components. In addition to Zaehle’s results, two studies reported effects of tDCS on AEP although the other studies presented different kinds of stimuli in their studies (Heimrath et al., 2016b; Impey et al., 2016). Heimrath et al. (2016b) investigated effects of tDCS on voiced and non-voiced syllables with stimulation located bilaterally at the AC. They report higher P50 amplitudes for syllables presented after anodal stimulation compared to sham and cathodal stimulation. TDCS did not affect N100 amplitudes, N100 latencies, or P50 latencies. Further, Impey et al. (2016) applied tDCS over the left AC to investigate effects on MMN. Their results also indicate that tDCS did not affect N100 amplitudes or latencies.

Taken together, only one study investigated the impact of tDCS on excitability of the AC in a systematic manner (Zaehle et al., 2011), and besides an increase in P50 amplitude (Heimrath et al., 2016b), results of the studies with a different type of stimulus are not consistent with the results by Zaehle et al. (Heimrath et al., 2016b; Impey et al., 2016). Therefore, we aimed to examine the effects of tDCS on the left posterior temporal cortex, thereby targeting the auditory cortex excitability, in more detail. The effectiveness of tDCS on processing in the AC is particularly interesting in the context of tDCS as a potential treatment option for electrophysiological parameters and symptoms of psychiatric diseases, e.g., lower N100 or auditory verbal hallucinations in schizophrenia (Ford et al., 2001a,b,c,d; Hubl et al., 2007; Li et al., 2016; Ponde et al., 2017; Gupta et al., 2018).

**FIGURE 1:**
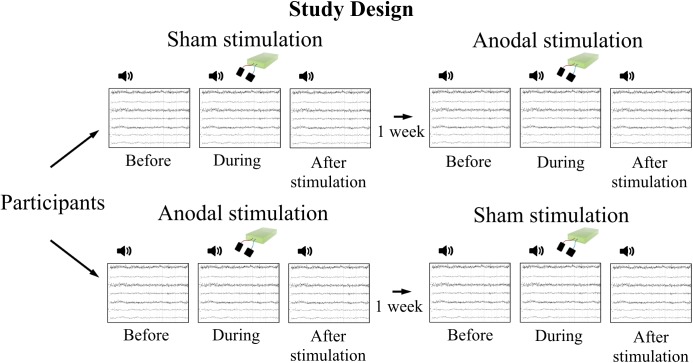
Study design. Participants attended two measurement sessions with 1 week in between to avoid carry-over effects of stimulation. Order of stimulation was assigned randomly. EEG was recorded during tone presentation at three time points per session: before, during, and after tDCS, respectively.

Zaehle et al. (2011) compared effects of stimulation types (anodal, cathodal, sham) *after* stimulation, we here extended this protocol by assessing AEP at three time points before, during, and after stimulation (Figure 1). We refrained from application of cathodal stimulation, as expectations of effects are not entirely clear for behavior in other than the motor domain (Jacobson et al., 2012). In line with earlier research (Zaehle et al., 2011; Heimrath et al., 2016b), we expected to see an increase of P50 amplitude, N100 amplitude, and N100 latency. In addition to P50 and N100 components, we investigated effects of tDCS on the P200, another positive auditory component about 200 ms after stimulus onset (Picton et al., 1974).

## Materials and Methods

### Participants

We included 24 healthy participants (21 female) between 18 and 64 years (*M* = 26.4, *SD* = 3.39). Concerning power, a sample size of 21 would be required to achieve a power of 0.8 with an effect size of 0.275 that we estimated based on prior work and α of 0.05. All participants were right-handed (Oldfield, 1971) and did not report any history of psychiatric or neurological disorders, or hearing impairment.

The study was approved by the Ethics Committee of Canton Bern (KEK-BE-2016-01741) and conducted in accordance with the Declaration of Helsinki. All participants provided written informed consent.

### Procedure

Measurements took place in a crossover design on 2 days with 1 week in between to avoid carry-over effects of stimulation (Figure 1). The order of stimulation (anodal, sham) was assigned randomly and stimulation applied in a double-blind design. Electroencephalography (EEG) was recorded before (as baseline), during, and after stimulation, while participants performed a passive listening task. One recording comprised presentation of 400 stimuli (tones), 200 stimuli twice with a 30 s break in between. To minimize habituation effects due to repetition of the same stimulus for many times, 200 tones were presented in advance of baseline EEG recordings as a pre-recording training.

### Acoustic Stimulation

Stimulus tones were generated using the cogent 2000 toolbox (version 1.32^1^, RRID:SCR_015672) for Matlab environment (version R2012a, The Mathworks, Inc., Natick, MA, United States, RRID:SCR_001622). Pure sinusoidal tones of 200 ms duration and 1000 Hz frequency were presented using Panasonic Technics SB-CS6 loudspeakers (Panasonic Corporation, Osaka, Japan) with an intensity of 65 dB and inter-stimulus intervals were jittered between 900 and 1100 ms.

### EEG Recordings

**FIGURE 2:**
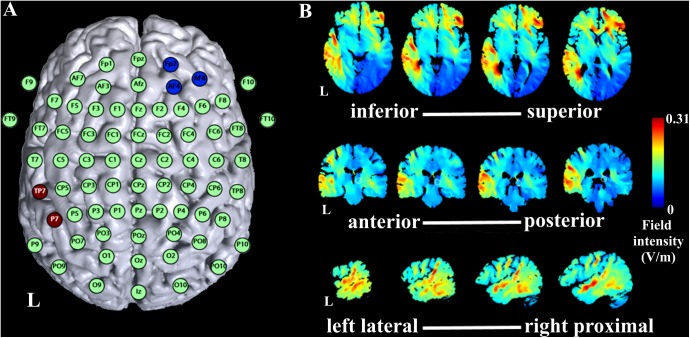
Simulation of tDCS current flow. **(A)** Montage of tDCS electrodes with anode over TP7 and P7 of the international 10–20 EEG system and reference electrode over Fp2, AF4, and AF8. **(B)** Simulation of 1 mA current flow with the montage of the current study with axial slices in the direction inferior to superior of the brain presented from left to right in the upper row of the figure, coronal slices in the direction of anterior to posterior in the brain presented from left to right in the middle row of the figure, and sagittal slices in the direction left lateral to right proximal in the left hemisphere of the brain presented from left to right in the lower row of the figure. L indicates left hemisphere.

EEG signal was recorded in a shielded room using a digital EEG amplifier system (BrainAmp DC amplifiers, Brain Products GmbH, Gilching, Germany, RRID:SCR_009443) and software (BrainVision Recorder, version 1.20.0601, Brain Products GmbH), filtered between 0.016 Hz and 1000 Hz with a sampling rate of 2500 Hz. Fifty-six passive Ag/Cl EEG electrodes were mounted on the scalp according to the international 10–20 EEG system with CPz as reference (Figure 2A). The ground electrode was placed at AFz. TDCS electrode montage required exclusion of electrode signals of Fp2, AF4, AF8, F6, TP9, TP7, CP5, P7, P5, PO7. Two additional electrodes on the outer canthi recorded electrooculograms of both eyes. All impedances were kept below 10 kΩ.

### TDCS Procedure

Stimulation was applied using a battery driven constant current stimulator (eldith, NeuroConn GmbH, Ilmenau, Germany) with the active 5 cm × 7 cm rubber electrode positioned over TP7 and P7 according to the international 10–20 EEG system, and the reference 5 cm × 5 cm rubber electrode over Fp2, AF4, and AF8 (Figure 2A). Impedances were kept below 10 kΩ. Stimulation montage was determined based on simulations with the software HD-Explore (Soterix Medical Inc., New York, NY, United States) aiming at stimulation of the AC (Figure 2B). For anodal stimulation, 1 mA was applied for 20 min (1 s fade in/out). For sham stimulation, the stimulator stopped stimulation after 30 s. Awareness of stimulation order was at 58% (14 out of 24 participants correctly identified order of stimulation), which is not significantly different of chance level, tested with a binomial test (*p* = 0.54).

### Data Analyses

#### Preprocessing

Offline preprocessing of EEG data was performed with the EEGLAB toolbox (version 14.1.1, Schwartz Center for Computational Neuroscience, La Jolla, CA, United States^2^) for Matlab environment (version R2017a). To remove eye blink-related artifact, we performed an independent component analysis (ICA) for recordings with stimulator on and off separately after band pass filtering to 1–30 Hz (Gebodh et al., 2017a,b). The estimated ICA matrices were then applied to 0.5–200 Hz band pass filtered raw data, thereby removing eye-blink related ICA components. Data was re-referenced to an average reference and segmented into epochs of 700 ms (200 ms pre-stimulus to 500 ms post-stimulus onset) with a baseline correction of 100 ms pre-stimulus interval. Epochs containing artifacts due to eye movements, muscular activity, or amplifier saturation were rejected manually. On average, 6% of trails were rejected and the average number of trials included into further analyses was not significantly different for the investigated conditions. Remaining epochs were averaged for every subject. Baseline-to-peak amplitudes and latencies for P50, N100, and P200, were identified by the automatic peak detection procedure implemented in BrainVision Analyzer at Cz where all subjects showed the typical AEP response to tone presentation.

For detection of potential outliers, i.e., subjects with a global difference in EEG signal compared to study population, a principal component analysis (PCA) and a topographical consistency test (TCT) were conducted with the Ragu toolbox (version March 07 2018^3^) for Matlab environment after downsampling the averaged data to 200 Hz (Koenig and Melie-Garcia, 2010; Habermann et al., 2018). The PCA allows for comparison of averaged single subject data to the mean of the study population. The TCT uses randomization statistics to evaluate similarity of subjects’ topographies at every time point. Both tests revealed no outlier for the current sample, so data of all subjects was included in further analyses.

#### AEP Analyses

Further analyses of amplitude and latency data were performed with SPSS (IBM Corp., IBM SPSS Statistics for Windows, Version 24.0.0, Armonk, NY, United States, RRID:SCR_002865). A repeated-measures analysis of variance (ANOVA) with factors stimulation (anodal, sham) and time point (pre, during, post-after stimulation) was conducted for P50, N100, and P200 amplitudes and latencies where data was normally distributed. Otherwise, non-parametric Wilcoxon Rank Sum tests with Bonferroni-Holm correction for multiple testing were calculated. All tests were accompanied by effect size calculations. In addition, we investigated effects of stimulation and time point on mean values in peak intervals by calculating repeated-measures ANOVAs with factors stimulation (anodal, sham) and time point (pre, during, post-after stimulation) for the three AEP. Furthermore, we were interested in potential responder/non-responder patterns in the data. We therefore calculated the differences of post-minus pre measurements in the anodal stimulation condition for all ERP amplitudes and latencies to account for changes of stimulation over time. Correlating these variables, we aimed to have a measure of global response to stimulation in one direction (excitation or suppression). Level of significance for all calculations was set to 0.05.

#### Topography Analyses

In addition to ERP analyses at single-electrode level, scalp field topographies were analyzed using a topographical ANOVA (TANOVA) (Murray et al., 2008; Habermann et al., 2018). The TANOVA employs non-parametric randomization statistics to explore global dissimilarity of topographical maps for different conditions at every time point (Lehmann and Skrandies, 1980; Murray et al., 2008; Koenig et al., 2011). In the current study, a TANOVA conducting 5000 permutations with factors stimulation (anodal, sham) and time point (before, during, after stimulation) was performed using the Ragu toolbox with a significance level of 0.05. As the TANOVA calculates dissimilarity of topographical maps at every time point, a correction for multiple testing over time, i.e., a duration correction, was applied to minimize potential false positive results (Koenig et al., 2011). Using statistics on the overall count of significant time points and duration of significant effects, Ragu provides the minimal duration of relevant effects separately for every effect, i.e., main or interaction effects. Results are reported for the time window of interest 0–300 ms after stimulus onset.

## Results

### AEP Analyses

**FIGURE 3:**
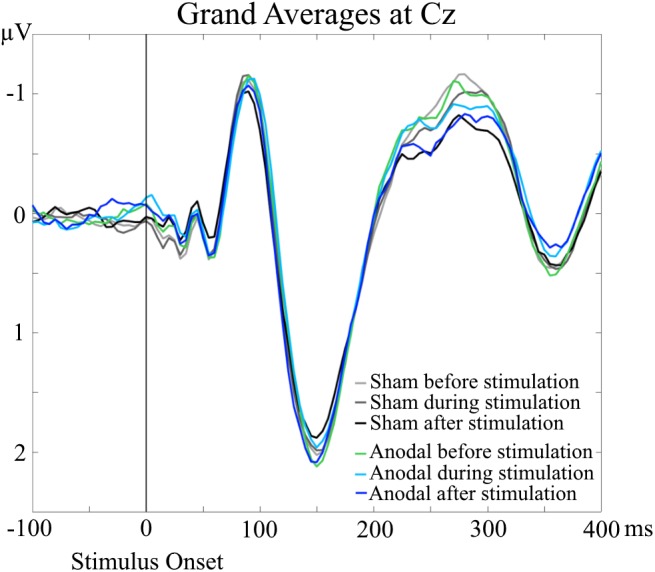
Grand Averages of AEP at Cz electrodes, separately for every condition. No significant differences were evident for P50, N100, and P200 amplitudes and latencies in the AEP analyses.

**FIGURE 4:**
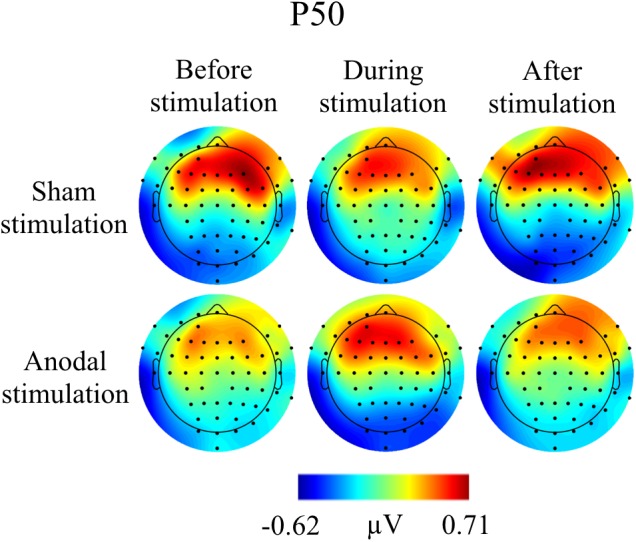
Topographies for grand average AEP of an interval 55–75 ms for P50 auditory component, separately for the different conditions. The peak interval was identified by grand average AEP.

**FIGURE 5:**
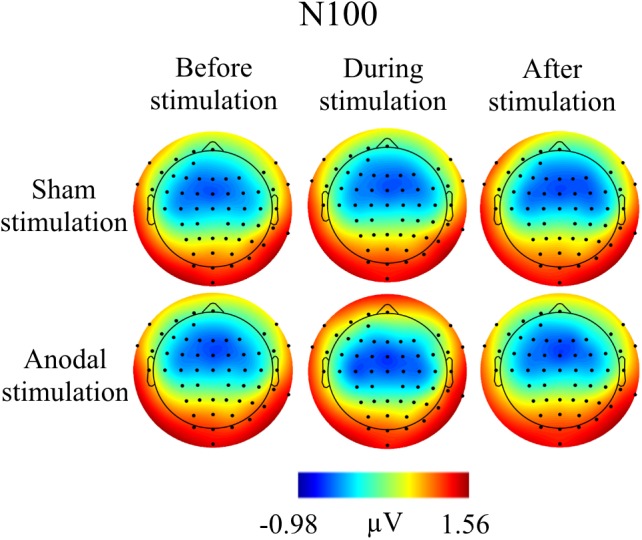
Topographies for grand average AEP of an interval 85–115 ms for N100 auditory component, separately for the different conditions. The peak interval was identified by grand average AEP.

**FIGURE 6:**
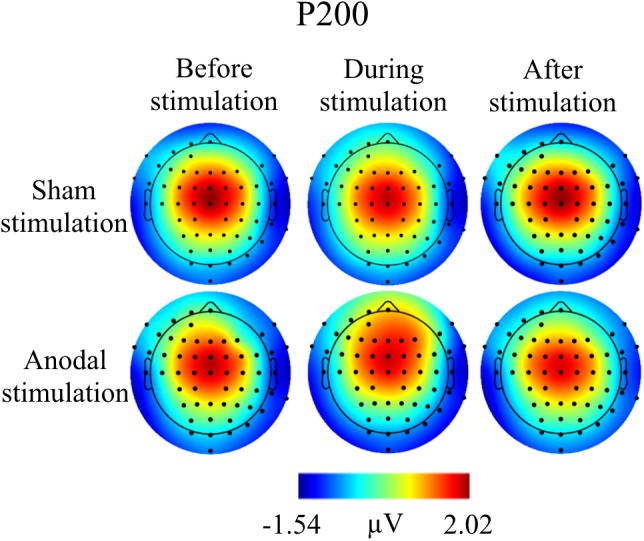
Topographies for grand average AEP of an interval 125–190 ms for P200 auditory component, separately for the different conditions. The peak interval was identified by grand average AEP.

Grand Averages of AEP for all conditions are presented in Figure 3, topographical maps for P50, N100, and P200 in Figures 4–6. Topographical maps show averaged data of peak intervals (55–75 ms for P50 peak, 85–115 ms for N100 peak, and 125–190 ms for P200 peak), identified using grand averages.

### P50

Mean amplitudes and latencies with standard deviations of P50 are reported for every condition in Table 1. Neither P50 amplitudes nor latencies were normally distributed, so several Wilcoxon Rank Sum tests were calculated for comparisons between time points and stimulation types (Table 2). None of the calculated tests survived the Bonferroni-Holm correction (global α = 0.05, α_1_ = 0.006, tested separately for amplitudes and latencies) and all show low effect sizes. In addition, the repeated-measures ANOVA on mean values in the peak interval did not show any interaction effect for factors stimulation and time point [*F*(2, 46) = 0.05, *p* = 0.95], nor an effect of factor stimulation [*F*(1, 23) = 1.55, *p* = 0.23], or time point [*F*(1, 23) = 0.86, *p* = 0.43].

**Table 1:** Mean amplitudes and latencies of P50 for the different conditions.

	Amplitudes (μV)	Latencies (ms)
Condition	*M* (*SD*)	*M* (*SD*)
Sham before	0.59 (0.52)	57.63 (6.68)
Sham during	0.57 (0.58)	57.78 (6.87)
Sham after	0.51 (0.64)	57.83 (7.94)
Anodal before	0.67 (0.66)	57.58 (7.69)
Anodal during	0.59 (0.64)	59.65 (6.27)
Anodal after	0.63 (0.57)	58.22 (7.59)

**Table 2:** Test statistics and effect sizes of Wilcoxon Rank Sum Tests for P50 latencies.

	Amplitudes			Latencies		
Comparison	*W*	*p*	*r*	*W*	*p*	*r*
Sham before vs. during	142.00	0.82	0.03	113.50	0.75	0.05
Sham during vs. after	119.00	0.38	0.13	127.00	0.99	0.00
Sham before vs. after	118.00	0.36	0.13	114.00	0.74	0.05
Anodal before vs. during	131.00	0.59	0.08	191.00	0.24	0.17
Anodal during vs. after	157.00	0.84	0.03	98.00	0.36	0.13
Anodal before vs. after	123.00	0.44	0.11	136.00	0.76	0.04
Before sham vs. anodal	170.00	0.57	0.08	107.50	0.78	0.04
During sham vs. anodal	153.00	0.93	0.01	158.50	0.53	0.09
After sham vs. anodal	185.00	0.32	0.14	125.50	0.73	0.05

*Reported p-values are uncorrected (significance level at Bonferroni-Holm-corrected global α = 0.05, α_1_ = 0.006).*

### N100

Table 3 shows mean amplitudes and latencies with standard deviations of N100 for every condition. Neither N100 amplitudes nor latencies were normally distributed. Thus, several Wilcoxon Rank Sum tests were calculated comparing the different time points and stimulation types for amplitudes and latencies separately (Table 4). None of the calculated tests showed a significant result with the Bonferroni-Holm correction (global α = 0.05, α_1_ = 0.006, tested separately for amplitudes and latencies) and all show low effect sizes. In addition, the repeated-measures ANOVA on mean values in the peak interval did not show any interaction effect for factors stimulation and time point [*F*(2, 46) = 0.05, *p* = 0.95], nor an effect of factor stimulation [*F*(1, 23) = 0.32, *p* = 0.58], or time point [*F*(1, 23) = 2.01, *p* = 0.15].

**Table 3:** Mean amplitudes and latencies of N100 for the different conditions.

	Amplitudes	Latencies
Condition	*M* (*SD*)	*M* (*SD*)
Sham before	-1.46 (0.97)	90.43 (8.73)
Sham during	-1.38 (0.93)	90.17 (7.89)
Sham after	-1.33 (0.83)	89.78 (10.81)
Anodal before	-1.44 (0.94)	89.35 (11.13)
Anodal during	-1.39 (1.05)	93.22 (9.51)
Anodal after	-1.43 (0.80)	89.18 (9.94)

**Table 4:** Test statistics and effect sizes of Wilcoxon Rank Sum Tests for N100 amplitudes and latencies.

	Amplitudes			Latencies		
Comparison	*W*	*p*	*r*	*W*	*p*	*R*
Sham before vs. during	166.00	0.65	0.07	123.00	0.79	0.04
Sham during vs. after	154.00	0.91	0.02	171.00	0.64	0.07
Sham before vs. after	183.00	0.35	0.14	141.50	0.81	0.04
Anodal before vs. during	159.00	0.80	0.04	181.50	0.19	0.19
Anodal during vs. after	146.00	0.91	0.02	114.00	0.30	0.15
Anodal before vs. after	144.00	0.86	0.02	165.00	0.41	0.12
Before sham vs. anodal	160.00	0.63	0.07	127.00	0.51	0.09
During sham vs. anodal	117.00	0.35	0.14	190.50	0.25	0.16
After sham vs. anodal	144.00	0.86	0.02	121.00	0.85	0.03

*Reported p-values are uncorrected (significance level at Bonferroni-Holm-corrected global α = 0.05, α_1_ = 0.006, separately for amplitudes and latencies).*

### P200

For P200, mean amplitudes and latencies with standard deviations are shown for every condition in Table 5. P200 amplitudes and latencies were not normally distributed. Several Wilcoxon Rank Sum Tests were calculated to compare amplitudes and latencies for different types of stimulation and time points. None of the tests for showed significant results after Bonferroni-Holm correction (global α = 0.05, α_1_ = 0.006, tested separately for amplitudes and latencies Table 6) and all show low effect sizes. In addition, the repeated-measures ANOVA on mean values in the peak interval did not show any interaction effect for factors stimulation and time point [*F*(2, 46) = 0.96, *p* = 0.39], nor an effect of factor stimulation [*F*(1, 23) = 0.04, *p* = 0.84], or time point [*F*(1, 23) = 0.30, *p* = 0.75].

**Table 5:** Mean amplitudes and latencies of P200 for the different conditions.

	Amplitudes	Latencies
Condition	*M* (*SD*)	*M* (*SD*)
Sham before	2.34 (0.68)	150.58 (12.15)
Sham during	2.25 (0.57)	150.10 (9.70)
Sham after	2.19 (0.74)	150.15 (13.44)
Anodal before	2.34 (0.73)	150.75 (9.95)
Anodal during	2.20 (0.58)	150.72 (8.53)
Anodal after	2.39 (0.93)	148.67 (8.75)

**Table 6:** Test statistics and effect sizes of Wilcoxon Rank Sum Tests for P200 amplitudes and latencies.

	Amplitudes			Latencies		
Comparison	*W*	*p*	*r*	*W*	*P*	*r*
Sham before vs. during	121.00	0.41	0.12	112.50	0.65	0.07
Sham during vs. after	125.00	0.48	0.10	135.00	0.93	0.01
Sham before vs. after	109.00	0.24	0.17	151.00	0.98	0.00
Anodal before vs. during	120.00	0.39	0.12	147.00	0.93	0.01
Anodal during vs. after	175.00	0.48	0.10	91.50	0.10	0.24
Anodal before vs. after	151.00	0.98	0.00	124.00	0.46	0.11
Before sham vs. anodal	141.00	0.80	0.04	148.00	0.95	0.01
During sham vs. anodal	132.00	0.61	0.07	166.00	0.65	0.07
After sham vs. anodal	186.00	0.30	0.15	125.00	0.69	0.06

*Reported p-values are uncorrected (significance level at Bonferroni-Holm-corrected global α = 0.05, α_1_ = 0.006, separately for amplitudes and latencies).*

### Response Pattern Analysis

As amplitudes and latencies were not normally distributed, we calculated the phi coefficients for all difference variables. With Bonferonni-Holm correction for multiple comparisons (global α = 0.05, α_1_ = 0.003), none of the phi coefficients showed a significant correlation of the variables (*p* > 0.08).

#### Topography Analyses

The two-way TANOVA did not show significant topological differences for the interaction effect between stimulation and time point for duration-corrected time intervals of 25 ms. For the main effect of time point, there was a significant difference in topographies in the time period about 240–305 ms after stimulus onset. No duration-corrected significant time periods (>30 ms) were found for the main factor of stimulation.

## Discussion

In the current study, we applied 20 min of anodal and sham tDCS over the left posterior temporal cortex in a double-blind crossover design. We measured AEP before, during, and after stimulation. In contrast to our hypotheses, we did not find any differences in P50 amplitudes for anodal and sham stimulation, precisely for neither of the investigated AEP amplitudes or latencies. Furthermore, no effects of tDCS were evident when comparing AEP amplitudes or latencies before, during, and after stimulation. In addition, the topographical analyses did not indicate topographical differences for the investigated conditions.

Particularly interesting about our results is the fact that we found no difference of AEP amplitudes and latencies for the different measurement time points before, during, and after stimulation. As earlier studies reported effects of anodal tDCS on AEP after stimulation (Zaehle et al., 2011; Heimrath et al., 2016b; Impey et al., 2016), we also expected to see differences at least after anodal compared to sham stimulation. Furthermore, neither the analysis on mean values in peak intervals, nor the investigation for general response patterns or the topographical analysis suggested a global effect of stimulation. Thus, no effects on global scalp fields are a sign for no difference in active sources in the different conditions (Koenig et al., 2011). Altogether, our results create doubt about the effectiveness of tDCS to functionally modulate auditory processing, at least concerning low-level processing of acoustic stimuli. However, the possibility of a very subtle effect only observable in a larger sample cannot be excluded although we included a reasonable number of subjects to expect a potential effect on AC excitability by tDCS.

An interesting result of the TANOVA was the main effect of time point for the time interval of 240–305 ms. For the grand averages, the amplitudes of the AEP in this particular time interval occurred to be different (Figure 3). The amplitudes appeared to decrease over time, independent of stimulation condition, which resembles a habituation effect over the progress of the measurement session independent of stimulation application (Butler, 1968; Maclean et al., 1975; Sambeth et al., 2004; Gandelman-Marton et al., 2010). To minimize a potential habituation effect, we presented additional stimuli in advance of the baseline EEG recordings. We only saw the habituation effect in the topographical analysis and only in a late time interval after stimulus onset. This is in line with the results of Bruin et al. (2000) who found a faster habituation for visual N100 than for P300 (with just a trend at Cz). Thus, our pre-measurement training was sufficient to suppress a habituation effect for N100, but not for the later component that we saw in the topographical analysis.

There is substantial discrepancy among the results of our study and previous studies, and the difference in results could be explained by differences in stimulation protocols and study design. In the next paragraphs, we will discuss in more detail three parameters that crucially affect tDCS effects, i.e., montage, stimulation intensity and duration, as well as stimulation protocol, i.e., the conditions to be compared.

First, one difference in study protocols between our and previous studies is the tDCS electrode montage. Zaehle et al. (2011) employed two montages similar to montages used in earlier studies (Fregni et al., 2006; Vines et al., 2006) with the active electrode either located temporally (over T7 of the 10–20 EEG system) or temporo-parietally (over CP5) and the reference electrode located over the contralateral supraorbital area (Zaehle et al., 2011). In the current study, we determined the montage based on simulations by a current flow simulation software (Figures 2A,B). Following the simulations, we concluded that the temporo-parietal location (over TP7 and P7) was optimal to target the AC. Given the differences Zaehle et al. (2011) found for the two montages they compared, one might conclude that even a small change of the position of the active electrode affects the stimulation outcome considerably. Thus, the shift of the active electrode to a more parietal location in the current study, although expectedly optimal for our purposes, might have accounted for our unexpected results. However, we cannot rule out a potential effect of cathodal stimulation at the parietal location due to our study design, which is a limitation of our study.

Second, there is a high variability in stimulation durations and intensities among studies, which make a direct comparison of results difficult. Zaehle et al. (2011) applied 1.25 mA for 11 min, Heimrath et al. (2016b) applied 1.5 mA for about 22 min, while we used 1 mA for 20 min. As the optimal parameters for tDCS efficacy still have to be determined, researchers will need to accomplish a high standard for their methods, and this includes the choice of optimal stimulation parameters. The currently high variability among stimulation parameters coupled with diverging research outcomes might preferably result in a consensus, as probably not all variables can be set optimally. In the case of our study, we had to make a compromise by choosing a smaller tDCS reference electrode in order to minimize data loss during concurrent tDCS and EEG measurements for better EEG electrode coverage. This led to a higher current density at the smaller tDCS reference electrode (Nitsche and Paulus, 2000). The current density for the active electrode, however, was still over the minimum threshold to expect an effect (Nitsche and Paulus, 2000). Furthermore, we included this difference in current density as a factor in current simulations prior to study measurements (see Figure 2).

Last, the study designs, i.e., the stimulation conditions compared, differ across studies. Zaehle et al. (2011) used a sequential design with anodal or cathodal stimulation following sham stimulation in two sessions. They did not report any effect of sham stimulation on AEP, because they focused on the comparison of anodal, cathodal, and sham stimulation after stimulation application. In contrast, our focus was on the comparison of anodal and sham stimulation over the time course before, during, and after stimulation. Hence, the differences in the study design further complicate the comparison of the results between studies.

Our results indicate a need of method optimization, i.e., to test and compare different stimulation parameters to gain an overview of parameters that are effective. Thus, further studies considering other auditory tasks, electrodes montages, application of cathodal stimulation, or variation of other stimulation parameters are warranted to investigate potentially specific or subtle effects of tDCS we might have missed with our design. Particularly for the application of tDCS in auditory processing or psychiatric symptoms (e.g., a lowered N100 or decreased auditory verbal hallucinations in schizophrenia; Ford et al., 2001a,b,c,d; Hubl et al., 2007), the determination of the optimal parameters to effectively target the AC would be essential.

## Conclusion

In conclusion, when applying anodal and sham tDCS on the left posterior temporal cortex to target the excitability of AC, we unexpectedly did not observe any significant effect of stimulation on AEP, along with no effect of time point when comparing the data before, during, and after stimulation. As the topographical analysis also did not indicate any differences between conditions, we doubt that the stimulation parameters we used in our tDCS protocol are effective to modulate auditory processing on a local scale, i.e., changing AEP on single-electrode level, or global scale, i.e., whole-topography changes. Additional investigations are warranted to explore effective parameters for tDCS to modulate auditory processing for applications in both, healthy subjects, and patients with psychiatric symptoms, e.g., auditory verbal hallucinations.

## Data Availability

The raw data supporting the conclusions of this manuscript will be made available by the authors, without undue reservation, to any qualified researcher.

## Ethics Statement

This study was carried out in accordance with the recommendations of the Ethics Committee of Canton Bern with written informed consent from all subjects. All subjects gave written informed consent in accordance with the Declaration of Helsinki. The protocol was approved by the Ethics Committee of Canton Bern (KEK-BE-2016-01741).

## Author Contributions

LM, YM, and TD designed the study. KK, MG, and YM conceptualized and performed data analysis. KK collected the data and wrote the first draft of the manuscript. All authors contributed to discussion about interpretation of the results, revised, and approved the final version of the submitted manuscript.

## Conflict of Interest Statement

The authors declare that the research was conducted in the absence of any commercial or financial relationships that could be construed as a potential conflict of interest.

## Footnotes

1 www.vislab.ucl.ac.uk

2 https://sccn.ucsd.edu/eeglab/index.php, RRID:SCR_007292

3 www.thomaskoenig.ch/index.php/software/ragu
